# Characteristics of patients infected with *Clostridioides difficile* at a Saudi Tertiary Academic Medical Center and assessment of antibiotic duration

**DOI:** 10.1186/s13099-021-00405-9

**Published:** 2021-02-17

**Authors:** Khadijah M. Alammari, Abrar K. Thabit

**Affiliations:** Pharmacy Practice Department, Faculty of Pharmacy, King Abdulaziz University, 7027 Abdullah Al-Sulaiman Rd, Jeddah, 22254-2265 Saudi Arabia

**Keywords:** *Clostridioides difficile*, *Clostridium difficile*, Antibiotics, Saudi arabia

## Abstract

**Background:**

*Clostridioides difficile* infection (CDI) is a common hospital-associated diarrhea. Several antibiotics commonly associate with CDI; however, limited data are available on the duration of exposure prior to CDI. Moreover, studies on the characteristics of CDI patients in Saudi Arabia are limited. Therefore, this study aimed to characterize CDI patients identified over 10 years and assess antibiotic days of therapy (DOT) prior to CDI.

**Methods:**

This was a retrospective descriptive analysis of CDI patients at a Saudi tertiary academic medical center between December 2007 and January 2018. Patients characteristics, prior exposure to known CDI risk factors, and DOT of antibiotics prior to CDI incidence were assessed.

**Results:**

A total of 159 patients were included. Median age was 62 years. Most cases were hospital-acquired (71.1%), non-severe (44.7%), and admitted to medical wards (81.1%). Prior exposure to antibiotics and acid suppression therapy were reported with the majority (76.1 and 75.5%, respectively). The most frequently prescribed antibiotics were piperacillin/tazobactam, ceftriaxone, meropenem, and ciprofloxacin with median DOTs prior to CDI incidence of 14 days for the β-lactams and 26 days for ciprofloxacin. The distribution of DOT was significantly different for piperacillin/tazobactam in different units (*P* = 0.003) where its median DOT was the shortest in medical wards (11 days), and for ciprofloxacin among different severity groups (*P* = 0.013), where its median DOT was the shortest in severe CDI patients (11 days).

**Conclusion:**

Most patients in this study had hospital-acquired non-severe CDI and were largely exposed to antibiotics and acid suppression therapy. Therefore, such therapies should be revised for necessity.

## Background

*Clostridioides difficile* infection (CDI) is the most common cause of hospital-associated diarrhea [1]. Generally, the acquisition of CDI is categorized based on the exposure to the healthcare system into hospital-onset (HO-CDI), community-acquired (CA-CDI), and community-onset healthcare facility-associated (CO-HCFA) [1]. Some patients with a recurrent CDI episode who get exposed to the healthcare system were found to acquire a strain of CDI that is different from the index strain that caused the initial episode [2]. This finding adds to the evidence that one of the important modes of acquiring CDI is through hospitalization or exposure to healthcare by other means, such as regular hemodialysis or residence in nursing homes.

Certain risk factors are also known to be associated with CDI, such as exposure to antibiotics, older age, use of acid-suppressing agents, and use of antineoplastic agents [3, 4]. Identifying these risk factors in admitted patients can help predicting the risk of acquiring the infection; hence, decreasing the exposure to modifiable factors, such as antibiotics and acid suppression therapy. Some antibiotics or classes of antibiotics are linked to CDI more than others. Penicillins, cephalosporines, carbapenems, fluroquinolones, and clindamycin are associated with CDI incidence that is folds higher than other antibiotics [4–7]. Time from antibiotic exposure to CDI development was reported in two previous studies. One evaluated CDI incidence while patients were still on therapy, whereas the other evaluated the incidence after antibiotic therapy cessation [4, 6]. The studies found an exposure of as short as a few days to as long as three months post therapy discontinuation was followed by CDI.

Data from Saudi Arabia on the characteristics of CDI patients are very limited. The majority of cases reported from three studies had HO-CDI followed by lower rates of CA-CDI and CO-HCFA [8–10]. Antibiotic exposure within three months was found with 26 of 42 cases (61%) in one of the studies [8]. Other studies from the Middle East showed a similar prevalence pattern of CDI acquisition with antibiotic exposure (particularly fluoroquinolones, cephalosporins, and carbapenems) and proton pump inhibitors being the most reported factors predisposing CDI [11–15]. No additional CDI data from Saudi Arabia were found in the literature, as well as additional data on time to CDI incidence from antibiotic therapy initiation. Therefore, the objective of this study was to describe the characteristics of patients who acquired CDI that was confirmed by a laboratory test for *C. difficile* in a Saudi hospital. The study also aimed to define the duration of antibiotic exposure that preceded CDI incidence in these patients.

## Methods

### Study design and patients

This was a retrospective descriptive study on adult (≥ 18 years old) CDI patients admitted to King Abdulaziz University Hospital, a tertiary academic medical center in Jeddah, Saudi Arabia. All patients presented to the hospital with CDI during the period from December 2007 to January 2018 were included. The characteristics of these patients, prior exposure to known CDI risk factors at the time of CDI incidence, and the duration of exposure to different antibiotics prior to CDI incidence (expressed as days of therapy, DOT) during or prior to the admission were assessed. Patients with inconsistent medication administration record data were excluded. The study was approved by the Research Committee of The Unit of Biomedical Ethics of Faculty of Medicine, King Abdulaziz University, Jeddah, Saudi Arabia.

### Definitions

CDI was defined as positive toxin immunoassay in patients with diarrhea (≥ 3 loose stools within one day). Acquisition forms of CDI were defined according to the Infectious Diseases Society of America (IDSA) and the United States Centers for Disease Control and Prevention (CDC) guidelines [1, 16]. CA-CDI was defined as a CDI episode that occurs in a patient with no history of hospitalization within the previous 12 weeks and 48 h or less of hospitalization. HO-CDI was defined as CDI onset three days after admission (on or after day 4). If the symptoms started within 28 days after hospital discharge, the condition is termed CO-HCFA. CDI testing at our institution is done using IMMUNOQUICK Tox A/B (Biosynex, France), which has 88% sensitivity and 99% specificity [17]. According to the protocol of the microbiology laboratory at our institution, all formed stool samples are rejected; hence, only loose stool samples are accepted for CDI testing. Moreover, our hospital protocol mandates that stool specimens be transported to the microbiology laboratory as soon as possible within 1–2 h of collection to avoid potential degradation of *C. difficile* toxins. DOT was defined according to the CDC’s National Healthcare Safety Network as the aggregate sum of days for which any amount of a specific antimicrobial agent was administered to individual patients as documented in the electronic medical record [18].

### Statistical analysis

Data are presented using descriptive statistics as numbers, percentages, and median [interquartile range]. For the most used antibiotics, subgroup analyses using Mann–Whitney U test or Kruskal–Wallis test were carried out to determine the difference between the DOT of antibiotics in the subgroups. A *P* value of < 0.05 was considered significant. Statistical analysis was carried out using SPSS version 24.0 software (SPSS, Inc., Chicago, Illinois, USA).

## Results

Of 167 identified patients with CDI, 159 were included as 8 patients had inconsistent antibiotic administration records. Characteristics of included patients are shown on Table 1. Most of the patients acquired CDI while in the hospital (71.1%), and three-quarters had a prior antibiotic and acid suppression therapy exposure (76.1 and 75.5%, respectively). Of 113 HO-CDI patients, 100 (88.5%) were exposed to antibiotics prior to CDI incidence compared with 6 of 28 patients (21.4%) and 15 of 18 patients (83.3%) who developed CA-CDI and CO-HCFA CDI, respectively. A similar pattern was seen with the exposure to acid suppression therapy, where 95 (84.1%), 9 (32.1%), and 16 (88.9%) of HO-CDI, CA-CDI, and CO-HCFA CDI patients, respectively, received a proton pump inhibitor or an H_2_-receptor antagonist. Only a few patients were on cancer chemotherapy or had a history of a lower gastrointestinal disease, such as inflammatory bowel disease or diverticulitis. The proportion of patients that developed non-severe CDI was the largest (44.7%) followed by severe CDI (29.6%) and severe complicated/fulminant (25.8%).

**Table 1:** Patients characteristics

Characteristic	N (%) or Median [IQR]
Age (years)	62 (48–71)
Sex (male)	80 (50.3)
Hospital unit	
Medical	129 (81.1)
ICU	19 (11.9)
ED	11 (6.9)
Acquisition	
CA-CDI	28 (17.6)
HO-CDI	113 (71.1)
CO-HCFA	18 (11.3)
Severity	
Non-severe	71 (44.7)
Severe	47 (29.6)
Severe complicated (fulminant)	41 (25.8)
Time to CDI from admission, days^a^	7 (2–20)
Non-CDI antibiotic therapy	121 (76.1)
Number of antibiotics^b^	3 (1–5)
Cancer chemotherapy	22 (13.8)
Acid suppression therapy	120 (75.5)
Type of acid suppression therapy^c^	
Proton pump inhibitor	47 (29.6)
H_2_-receptor antagonist	2 (1.3)
Antacid	1 (0.6)
> 1 type	70 (44)
Lower gastrointestinal disease	14 (8.8)

^a^In hospitalized patients, excluding patients presented to the ED

^b^In the subset of patients who received antibiotics

^c^In the subset of patients who received acid suppression therapy

*CA-CDI* community-acquired *C. difficile* infection,* CDI* *C. difficile* infection,* CO-HCFA* community-onset healthcare facility-associated,* DOT* days of therapy,* ED* emergency department,* HO-CDI*, hospital-onset *C. difficile* infection,* ICU* intensive care unit,* IQR* interquartile range

Table 2 presents the frequency of use of antibiotics in a descending order with their median DOT. The most frequently prescribed antibiotics were piperacillin/tazobactam, ceftriaxone, meropenem, and ciprofloxacin with median DOTs prior to CDI incidence of 14 days for the β-lactams and 26 days for ciprofloxacin.

**Table 2:** Frequency of antibiotics and their days of therapy (DOT)

Antibiotic	N (%)	DOT (median [IQR])
All antibiotics	159 (100)	17.5 [12.25–26.5]
Piperacillin/tazobactam	62 (39)	14 [5.75–40.5]
Ceftriaxone	60 (37.7)	14 [5–43.25]
Meropenem	59 (37.1)	14 [7–26]
Ciprofloxacin	50 (31.4)	26 [8.75–56.5]
Cefuroxime	37 (23.3)	14 [7.5–44.5]
Gentamicin	18 (11.3)	18 [5.75–44]
Imipenem/cilastatin	19 (11.9)	11 [4–29]
Ceftazidime	17 (10.7)	14 [4.5–40]
Amoxicillin/clavulanic acid	15 (9.4)	10 [9–26]
Clarithromycin	14 (8.8)	11.5 [7.75–34.5]
Clindamycin	14 (8.8)	27 [9.75–58]
Trimethoprim/sulfamethoxazole	12 (7.5)	9.5 [4.25–62.25]
Cefazolin	12 (7.5)	13 [7.5–16.25]
Cloxacillin	10 (6.3)	37 [10.75–58.25]
Moxifloxacin	9 (5.7)	38 [10–61]
Azithromycin	7 (4.4)	26 [5–58]
Erythromycin	7 (4.4)	47 [3–56]
Rifampin	5 (3.1)	7 [5.5–36]
Amikacin	4 (2.5)	35 [10.75–54]
Ampicillin	4 (2.5)	6 [2–10.75]
Cefepime	4 (2.5)	17.5 [9–55.25]
Tigecycyline	4 (2.5)	30 [7–53.25]
Colistimethate sodium	3 (1.9)	26 [9–29]
Flucloxacillin	2 (1.3)	17–30^a^
Ofloxacin	2 (1.3)	38-49^a^
Doxycycline	1 (0.6)	3
Levofloxacin	1 (0.6)	21

^a^Range

**Fig. 1:**
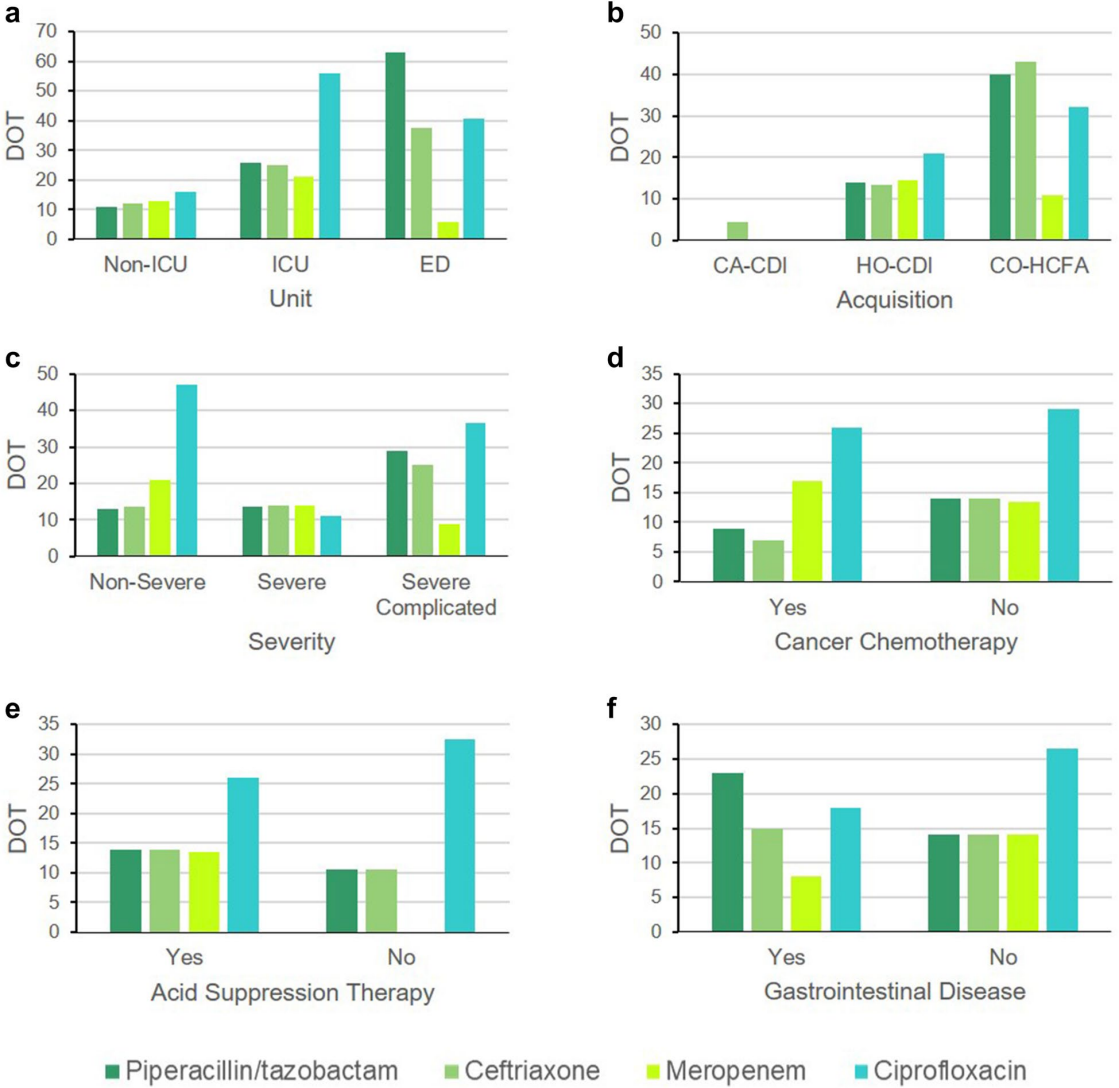
Median days of therapy of the four most used antibiotics in the subgroups. Significant difference was observed in the distribution of DOT of piperacillin/tazobactam (*P* = 0.003) between the hospital units, and of ciprofloxacin between severity groups (*P* = 0.013). CA-CDI, community-acquired *C. difficile* infection; CO-HCFA, community-onset healthcare facility-associated; DOT, days of therapy; ED, emergency department; HO-CDI, hospital-onset *C. difficile* infection; ICU, intensive care unit

In the subgroup analysis, the distribution of the four most commonly prescribed antibiotics largely followed the distribution of patients in the different categories as reported in Table 1. For example, since most of the patients during the study period were admitted to the medical ward, they represented the group that received most of the antibiotics. The DOT of these antibiotics appear in Fig. 1, where the distribution of DOT was significantly different for piperacillin/tazobactam in different units (*P* = 0.003). Counterintuitively, patients in non-intensive care unit (ICU) wards had the shortest antibiotic exposure to piperacillin/tazobactam prior to CDI development with a median DOT of 11 days. The distribution of DOT was also significant for ciprofloxacin but among the different severity groups where the shortest median DOT was reported in the group of patients who exhibited severe CDI (11 days; *P* = 0.013). Generally, in all other subgroups, shorter courses of meropenem preceded CDI compared with ciprofloxacin which were given for more days before CDI ensued as illustrated in Fig. 1; though, the differences were not statistically significant.

## Discussion

CDI is the greatest reason for hospital-associated diarrhea [1]. In fact, the incidence of *C. difficile* colonization in the first days of hospitalization ranges from 16 to 20%; however, it increases with longer hospital stay up to 45.4% [19, 20]. HO-CDI was consistently the major mode of CDI acquisition in our patient population despite a low overall incidence at our center where the cumulative 10-year incidence in adults was 7% only [21]. The reporting of CO-HCFA CDI cases also indicate that recent hospitalization can amplify the risk of CDI acquisition. In fact, one study found that exposure to healthcare can be associated with colonization of different *C. difficile* strain in patients previously infected with the pathogen [2]. On the other hand, CA-CDI incidence in our population was much lower at 17.6% followed by CO-HCFA CDI at 11.3% only. The occurrence of CA-CDI was reported in low-risk populations, including those in younger age groups, those with no recent exposure to antibiotic, acid suppressants, asymptomatic carriers, contaminated food or water, and hypervirulent strains, as well as those with no recent outpatient visits and who are not in close proximity to farms [22]. However, the presence of any of these factors in non-hospitalized patients is also linked to CDI development as reported in a study by Chitnis et al. who found that 64.1% of patients with confirmed CA-CDI received antibiotics in a previous outpatient visit [23]. These findings were consistent with our patient population that developed CO-HCFA CDI, but not with CA-CDI patients, where the majority of the former were exposed to antibiotic, acid-suppression therapy, or both.

Patients who are 65 years or older can be at five- to ten-fold higher risk for CDI, most likely with poor prognosis compared with younger patients [3, 5]. In our study, half of the patient population were above 62 years. Thus, many were already at risk for CDI acquisition. Nonetheless, the presence of other risk factors may have augmented the risk. For instance, exposure to antibiotics and acid suppression therapy have been reported in about three-quarters of the patients in our study. The latter, especially proton pump inhibitors and H_2_-receptor antagonists, is significantly associated with CDI development [24], and most patients who received acid suppression therapy in the current study received more than one kind, such as a proton pump inhibitor or an H_2_-receptor antagonist. Conversely, cancer chemotherapy was reported in a small fraction of patients (13.8%). This can be possibly attributed to the fact that our center is not an oncology specialized center. Nevertheless, if that was the case, more CDI patients would have been included since cancer chemotherapy is another known risk factor for CDI [4, 25]. Similarly, lower gastrointestinal diseases, particularly inflammatory bowel disease, can put patients at risk for CDI [26]. However, this was also reported in a small number of patients in this study (9.3%). Of all these CDI risk factors, studies from Saudi Arabia identified antibiotics and proton pump inhibitors as the major risk factors that were significantly associated with CDI development, which were the two most prevalent factors in our patient population [10, 27, 28].

It was not surprising that many CDI patients in our study were exposed to antibiotics, where a median of three antibiotics were used (collectively, but not necessarily concurrently) at a median total DOT of 17.5 days. Notably, the most frequently prescribed antibiotics in our study belong to culprit antibiotic classes, β-lactams and fluoroquinolones. Similar finding was reported in another study from Saudi Arabia that evaluated antibiotics use, where cephalosporins and fluoroquinolones were the most used antibiotics [8]. The prescribing pattern of antibiotics in Saudi Arabia included β-lactams, fluoroquinolones, and macrolides on top of the list [29, 30]. A similar pattern was reported in studies from other countries, such as Canada, Italy, India, Jordan, and Turkey [31–36]. However, a study from the United States showed a slight decrease in the rate of prescription of antipseudomonal β-lactams (except carbapenems which showed an increase) and fluoroquinolones in favor of narrow-spectrum β-lactams [37]. In our study, piperacillin/tazobactam and meropenem were most likely prescribed for empiric purposes. Both, as well as ceftriaxone and ciprofloxacin, were significantly associated with CDI in several previous studies. Results from two meta-analyses showed that β-lactams (of all classes), fluoroquinolones, and clindamycin were the antibiotics with the highest likelihood to increase risk of CDI [38, 39]. However, it is important to realize that the exposure to antibiotics alone may not be a factor, but also the duration of that exposure, as well. A short DOT to CDI was reported in a previous study, where an exposure of 6 days to cefazolin in 80 patients and of 8 days to cefepime in 186 patients preceded CDI [4]. Our study revealed that patients developed CDI after an antibiotic exposure of approximately 7 days. A study by Hengens et al. found that the probability of CDI incidence remains up to three months after the last dose of antibiotic [6].

As CDI incidence in Saudi Arabia was reported low from many institutions, especially from the community, it is presumed to have a slight impact on the healthcare system and the economy compared with the status in other countries where CDI incidence is high [9, 10, 21]. However, since CDI was mostly acquired in the hospital setting and given the predictability of its incidence based on the high prevalent risk factors of antibiotics and acid suppression therapy use, we hold that rapid discharge of stable patients to reduce hospital length of stay, appropriate antibiotic stewardship, and careful evaluation of the need for acid suppression therapy can further help limiting the development of CDI in vulnerable patients.

Our study has several strengths. It is one of very few studies to characterize the CDI population in Saudi Arabia, measures the prevalence of different ways CDI is contracted, and identifies the prevalence of known CDI risk factors. Additionally, the study spanned a period of 10 years and included detailed antibiotic utilization data. Nevertheless, the study was limited by a few factors. It was single-centered, which may limit the ability to generalize its results to other centers in Saudi Arabia. Also, there were some inconsistencies in the medication administration records of some patients that resulted in their exclusion. Moreover, patients who may have been hospitalized or undergone medical procedures in other facilities may not have been identified since such factors may increase the risk for *C. difficile* colonization. While a history of a lower gastrointestinal disease was recorded, history of gastrointestinal surgeries was not captured. Additionally, the only test utilized at our institution for *C. difficile* detection is toxin immunoassay with a reported sensitivity of 88%. This indicates that without the utilization of glutamate dehydrogenase test for initial screening or a second verification test (such as polymerase chain reaction or culture), roughly 12% of the tested population may have been missed and not included in the study due to false negative results.

## Conclusion

CDI is a common hospital-acquired infection. Many CDI patients in this study acquired it from the hospital setting in a non-severe form and were largely exposed to antibiotics and acid suppression therapy. Therefore, hospitalized patients should be assessed for the necessity of such therapies and discontinue them when deemed unnecessary after evaluating the overall clinical status before CDI may ensue.

## Data Availability

Data are available upon request from the authors.

## Abbreviations

CA-CDI: Community-acquired *Clostridioides difficile* infection
CDC: Centers for Disease Control and Prevention
CDI: *Clostridioides difficile* Infection
CO-HCFA: Community-onset healthcare facility-associated *Clostridioides difficile* infection
DOT: Days of therapy
ED: Emergency department
HO-CDI: Hospital-onset *Clostridioides difficile* infection
ICU: Intensive care unit
IDSA: Infectious Diseases Society of America
